# The Autophagy Related Gene CHAF1B Is a Relevant Prognostic and Diagnostic Biomarker in Hepatocellular Carcinoma

**DOI:** 10.3389/fonc.2020.626175

**Published:** 2021-01-26

**Authors:** Zunni Zhang, Yalong Zhang, Wuning Mo

**Affiliations:** ^1^Department of Clinical Laboratory, First Affiliated Hospital of Guangxi Medical University, Nanning, China; ^2^Department of Ultrasonic Medicine, First Affiliated Hospital of Guangxi Medical University, Nanning, China

**Keywords:** hepatocellular carcinoma, CHAF1B, prognostic, autophagy, biomarker

## Abstract

The role of autophagy in tumors is complex; based on known interactions between autophagy and hepatocellular carcinoma (HCC) pathogenesis, we hypothesized that autophagy-related genes (ARGs) may play an important role in HCC. The ARGs were obtained from the Human Autophagy Database and the Gene Set Enrichment Analysis. Based on the area under the curve (AUC) value >0.9 with p <0.0001 and Student’s T-test analysis with p <0.0001, differently expressed autophagy-related genes (DEARGs) with high diagnostic efficiency were found. Besides that, we searched in the PubMed database to find novel DEARGs associated with HCC. Then the DEARGs were validated in the GSE25097, GSE54236, GSE76427, GSE64041, Oncomine, and Human Protein Atlas datasets. Finally, survival analysis of CHAF1B in HCC and correlations of clinico-pathological characteristics and CHAF1B were performed based on the TCGA database. The mRNA and protein expression of 531 ARGs were analyzed and validated in eight independent cohorts. First, 18 DEARGs with high diagnostic efficiency were selected from the TCGA database, and nine of them were identified that had not previously been associated with HCC. These nine DEARGs were validated in the GSE25097, GSE54236, GSE76427, GSE64041, Oncomine, and Human Protein Atlas datasets. Additionally, we found that CHAF1B was associated with overall survival and relapse free survival at one, three, and five years. Furthermore, the univariate and multivariate Cox analyses revealed that the high expression of CHAF1B was an independent risk factor in HCC patients. This research demonstrated that CHAF1B was a novel diagnostic and prognostic signature biomarker that could be potentially useful for predicting the development of HCC and may provide new insights for HCC tumorigenesis and treatments.

## Introduction

Hepatocellular carcinoma (HCC) has a high mortality rate worldwide (1) and accounts for up to 90% of liver cancers (2). HCC is characterized by a poor prognosis and high aggressivity and is difficult to diagnose early (3). It is widely known that non-alcoholic fatty liver disease, hepatitis C virus, and alcohol abuse are strongly related to the occurrence of HCC (4–6). Liver biopsy is the most reliable method for diagnosing HCC; however, due to its invasiveness, this method is not suitable for large-scale liver cancer screening (7). Additionally, the underlying mechanisms of HCC are still poorly understood. Therefore, new biomarkers must be identified to enable the early diagnosis and an improved understanding of the underlying molecular mechanisms of HCC to reduce its mortality rate.

Autophagy is characterized by the degradation of damaged or aging organelles and proteins in lysosomes, which aid in the renewal of certain organelles and contribute to the metabolic needs of the cell itself (8, 9). The role of autophagy in cancer therapy, tumor progression, and tumorigenesis is complex and contradictory. In normal tissue, autophagy inhibits tumorigenesis by limiting cell proliferation, maintaining homeostasis, ensuring genomic integrity, and repairing damaged DNA (10). In tumor tissue, autophagy mainly promotes the survival of tumor cells and malignant tumor progression (11–14). Autophagy-related genes (ARGs) play an important role in the process of autophagy (15). Previous research has revealed that ARGs may act as novel biomarkers of a variety of cancers (16–18). However, the role of the entire subset of ARGs in the prognosis of HCC has not yet been investigated.

In this study, we evaluated the protein and RNA expression levels of ARGs from four different public databases. Furthermore, a combination of survival rates, clinico-pathological features, and Cox regression analyses revealed that chromatin assembly factor 1, subunit B (CHAF1B) was a novel diagnostic and prognostic signature biomarker in HCC patients.

## Materials and Methods

**Figure 1:**
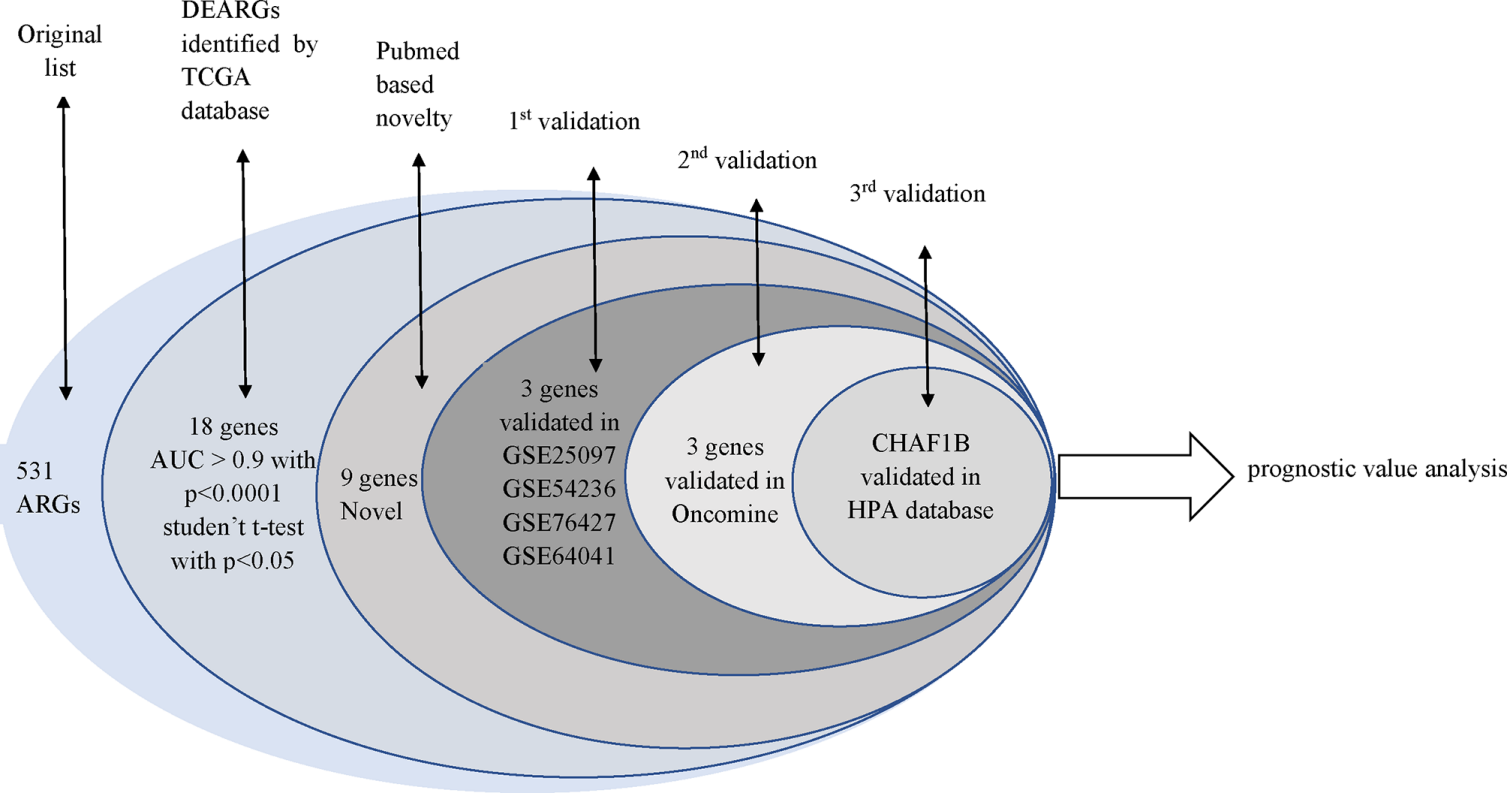
The flowchart of this study.

In total, 531 overlapped ARGs were obtained from the Human Autophagy Database (HADb, http://autophagy.lu), and GO AUTOPHAGY gene sets were acquired from the Gene Set Enrichment Analysis (GSEA, https://www.gsea-msigdb.org/gsea/index.jsp) in May 2020. The flowchart of this study was exhibited in **Figure 1**.

### The Selection of DE-ARGs Based on the TCGA Database: HCC *vs* Healthy Samples

From the Cancer Genome Atlas database (TCGA, https://portal.gdc.cancer.gov), we obtained the RNA-seq data of 49 normal and 370 HCC liver tissue samples. The expression of 531 ARGs was evaluated using the Student’s t test, and differentially expressed autophagy-related genes (DEARGs) were identified based on p <0.0001. Next, a receiver operating characteristic (ROC) analysis was carried out to assess the effectiveness of the given DEARGs in differentiating HCC from healthy samples. Significant area under the curve (AUC) values ranged from 0.5 to 1; the closer the AUC is to 1, the stronger the discrimination ability.

### The Selection of Unreported DEARGs in HCC

The DEARGs obtained from the TCGA database with an AUC >0.9 and a p <0.0001 were then searched in the PubMed database (https://www.ncbi.nlm.nih.gov/pubmed) on 27 May 2020 to determine whether they were significantly associated with HCC. To reduce the chances of false-negative results, we searched in “ALL fields.” The DEARGs that had not been reported to co-occur with HCC were regarded as novel biomarkers in the HCC field and were therefore selected for further analysis.

### The Validation of DEARGs

First-round validation: The gene expression of four datasets (GSE25097, GSE54236, GSE76427, and GSE64041) including 524 HCC and 446 control samples were downloaded from the Gene Expression Omnibus database (GEO, https://www.ncbi.nlm.nih.gov/gds). The relevant differentially expressed genes (DEGs) in HCC and control samples were identified according to p <0.0001. The cross DEGs among these four datasets were visualized using VENNY (version 2.1.0, https://bioinfogp.cnb.csic.es/tools/venny/index.html). Finally, the expression levels of three DEARGs in HCC patients that were substantially different from those in the control samples were found in the two databases of TCGA and GEO. These three DEARGs were selected for further validation.

Second-round validation: The Oncomine database (https://www.oncomine.org/resource/main.html) was utilized to validate the mRNA expression levels of previously verified DEARGs. The “Chen Liver” dataset contains 75 liver and 101 HCC samples, while the “Wurmbach Liver” dataset contains 75 total samples (10 liver and 35 HCC samples). The cut-off values were set as a fold change (FC) >1.2 and a p <0.05.

Third-round validation: The protein expression levels of the three previously identified and verified DEARGs were obtained from the Human Protein Atlas database (HPA, https://www.proteinatlas.org). Three healthy liver and 21 HCC tissues were retrieved. The cell staining intensity score revealed the following: no staining intensity was 0, medium staining intensity was 1, and high staining intensity was 2. The numbers of positive cells were assessed as follows: ≤0% scored 0; 1–6% scored 1; 6–25% scored 2; 26–75% scored 3; and 76–100% scored 4. The product of the cell staining intensity score and the score of the quantity of positive cells that ranged from 0 to 4 were judged as staining negative; all other scores were regarded as positive (score: 6–8).

### Survival Analysis

A Kaplan–Meier plot was used for the survival analysis. The patients were divided into a high and a low expression group by the median cut-off of verified DEARGs. The hazard ratio (HR) values with 95% a confidence interval (CI) and a logrank p-value were calculated. A logrank p <0.05 was considered statistically significant.

### A Comparison of the Expression of CHAF1B According to Clinico-Pathological Characteristics

The clinical data and mRNA expression data of 364 HCC patients based on the TCGA were obtained from the cBioPortal for Cancer Genomics (https://www.cbioportal.org). These patients were divided into high and low expression groups using the median expression level of CHAF1B.

### Protein–Protein Interaction Network and Enrichment Analysis

To identify the genes that interact with CHAF1B, a PPI network was performed using a STRING database (https://string-db.org) based on the criterion that medium confidence = 0.4. All the interacting genes were included in the Gene Ontology-Biological Process (GO-BP) and the Kyoto Encyclopedia of Genes and Genomes (KEGG) pathway enrichment analyses based on the ClueGO plug-in in Cytoscape (v. 3.7.2), and p <0.05 was set as the threshold of significant enrichment.

### Statistical Analysis

The Statistical Product and Service Solutions (SPSS), version 24.0, was used for the statistical analysis. Student’s t test was utilized to analyze the differences between the two variables, while the chi-square test was employed to assess the relationship of the expression of the CHAF1B and clinico-pathological characteristics. The ROC curve analysis generated by Medcalc was used to assess the diagnostic efficacy of ARGs. Univariate and multivariate cox analyses were performed to analyze the factors associated with the overall survival (OS). P <0.05 (two tailed) was considered statistically significant.

## Results

### The Selection of DEARGs From the TCGA Database

A total of 202 ARGs had strongly significant differences between the HCC and control samples based on the TCGA database that served DEARGs (p < 0.0001), and 18 of these 202 DEARGs exhibited a high diagnostic efficiency with an AUC >0.9 and p <0.0001. The PubMed search was performed on 27 May 2020. We found that nine of the DEARGs including SNAP-associated protein (SNAPIN), vacuolar protein sorting 33 homolog A (VPS33A), phosphofurin acidic cluster sorting protein 2 (PACS2), calpain 10(CAPN10), cathepsin A (CTSA), LSM4 homolog (LSM4), vacuolar protein sorting 28 homolog (VPS28), zinc finger with KRAB and SCAN domains 3 (ZKSCAN3), and CHAF1B had not been reported to be associated with HCC; they served as novel candidate HCC biomarkers and were used for further validation (**Table 1**).

**Table 1:** The expression and ROC analysis of ARGs in TCGA database.

No	Gene symbol	Mean expression in HCC	Mean expression in control	p-value	AUC	ARG reported for HCC in PubMed
1	BIRC5	1338.476	54.1224	<0.0001	0.973	≥1
2	CDKN2A	881.9568	38.5714	<0.0001	0.951	≥1
3	CLN3	1761.56	612.4694	<0.0001	0.941	≥1
4	RAB24	452.9378	155.8163	<0.0001	0.937	≥1
5	LSM4	5,100.578	1861	<0.0001	0.936	0
6	PEA15	6082.446	1,897.633	<0.0001	0.935	≥1
7	SNRPB	7362.876	2,341.878	<0.0001	0.932	≥1
8	SNAPIN	1,500.478	653.102	<0.0001	0.923	0
9	CHAF1B	369.5108	40.9388	<0.0001	0.922	0
10	HSP90AB1	53311.34	1,8807.98	<0.0001	0.921	≥1
11	TP73	179.7297	11.7347	<0.0001	0.919	≥1
12	CDC37	6,337.065	3,175.347	<0.0001	0.915	≥1
13	VPS33A	1,028.641	436.1837	<0.0001	0.914	0
14	ZKSCAN3	415.427	105.9796	<0.0001	0.912	0
15	PACS2	1,691.941	690.2245	<0.0001	0.909	0
16	CTSA	16,048.41	6072.959	<0.0001	0.908	0
17	CAPN10	611.6297	241.0612	<0.0001	0.905	0
18	VPS28	8,330.284	3,233.408	<0.0001	0.904	0

Only showing the DEARGs with AUC>0.9 and p<0.0001. The DEARGs reported for HCC in PubMed was “0” were served as novel biomarkers.

**Figure 2:**
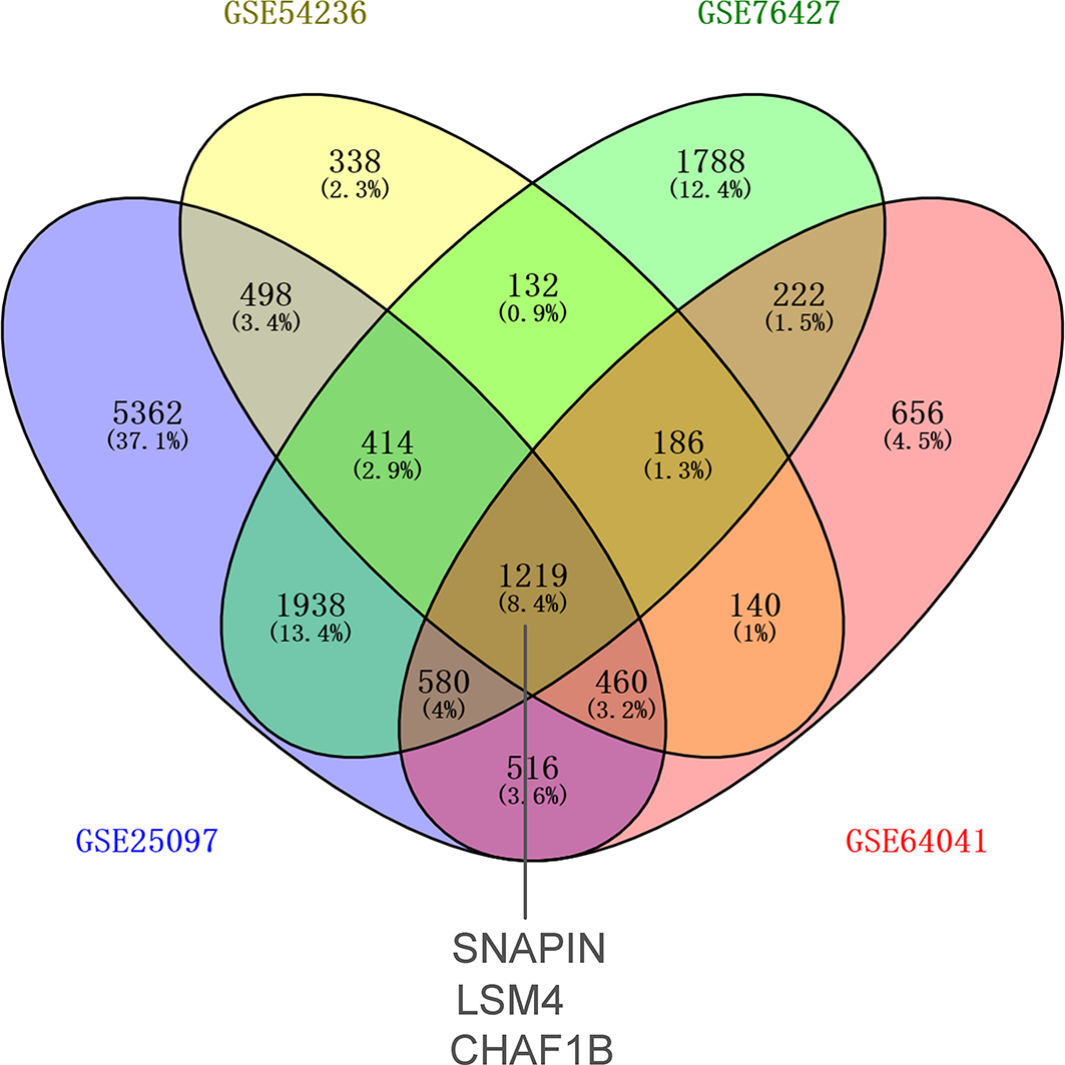
The intersecting differentially expressed genes in GEO datasets. Three of them were ARGs.

First-round validation: The nine DEARGs selected from the previously described analysis were validated in the GEO database. In total, 1,219 common differential expression genes (DEGs) were found based on GSE25097, GSE54236, GSE76427, and GSE64041. Three of these 1,219 DEGs were ARGs: SNAPIN, CHAF1B, and LSM4 (**Figure 2**).

Second-round validation: The three ARGs from the first-round validation were validated in the Oncomine database. All three ARGs have differential significance between HCC and control samples based on Chen Liver (SNAPIN: FC = 1.589, P = 1.38E-15; CHAF1B: FC = 1.311, P = 0.023; LSM4: FC = 1.735, P = 2.47E-15) and Wumbach Liver (SNAPIN: FC = 1.39, P = 0.002; CHAF1B: FC = 1.223, P = 0.044; LSM4: FC = 1.642, P = 7.24E-4) analyses (**Table 2**).

**Table 2:** The expression of DEARGs in Oncomine database.

	Gene symbol	FC	P-value
Chen Liver	SNAPIN	1.589	1.38E-15
	LSM4	1.735	2.47E-15
	CHAF1B	1.311	0.023
Wumbach Liver	SNAPIN	1.39	0.002
	LSM4	1.642	7.24E-4
	CHAF1B	1.223	0.044

**Figure 3:**
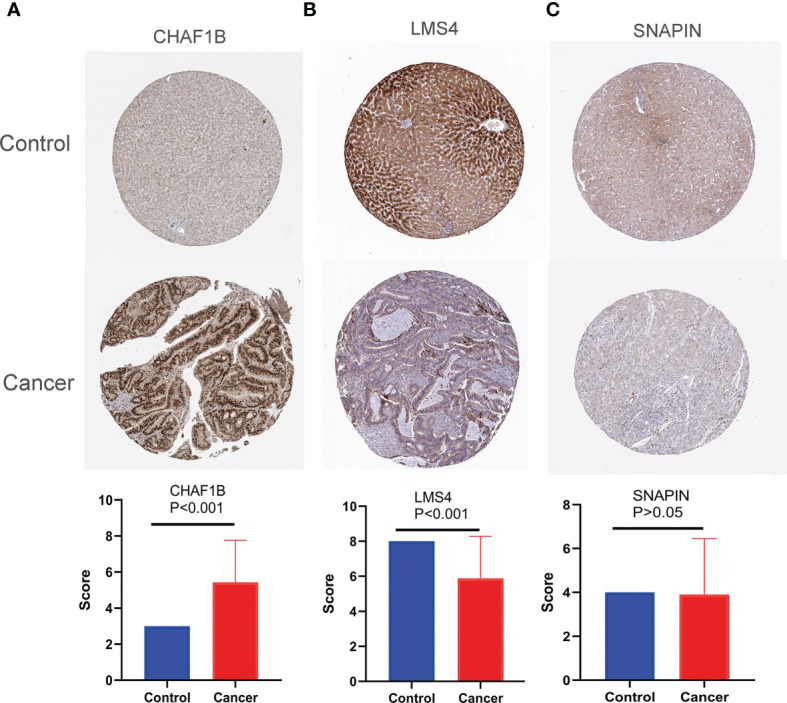
The protein expression of DEARGs. CHAF1B **(A)**, LSM4 **(B)**, SNAPIN **(C)**.

Third-round validation: Based on the HPA database, the protein expression levels of SNAPIN, CHAF1B, and LSM4 were evaluated according to the scoring criteria described in the “*Materials and Methods*” section. Consistent with the mRNA level, the protein level of CHAF1B is significantly increased in HCC tissues (p < 0.001), while LSM4 is decreased in HCC tissues (p < 0.001), and the expression levels of SNAPIN were unchanged (p = 0.8395) when compared with normal tissues (**Figure 3**).

### Prognostic Significance

**Figure 4:**
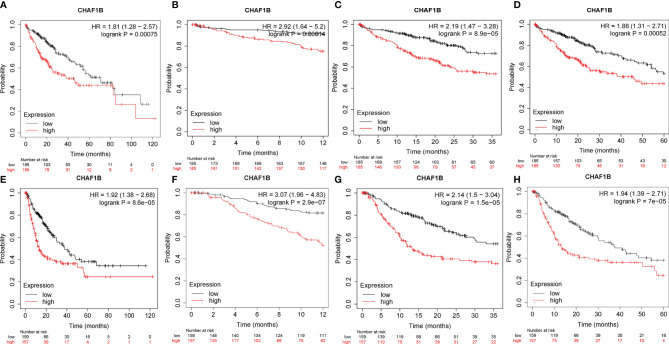
Correlation between CHAF1B and overall survival (OS) and recurrence-free survival (RFS) in HCC patients. Overall survival **(A)**, one-year OS **(B)**, three-year OS **(C)** and five-year OS **(D)**. RFS **(E)**, one-year RFS **(F)**, three-year RFS **(G)** and five-year RFS **(H)**.

The survival analysis of CHAF1B was investigated using the TCGA database. As shown in **Figure 4**, the high expression of CHAF1B in HCC was not only significantly related with poor OS (HR: 1.81 and p = 0.00075) but was also correlated with poor one-year (HR: 2.92, p = 0.00014), three-year (HR: 2.19, p = 8.9E-5), and five-year (HR: 1.88, p = 0.00052) OS. Additionally, the relapse free survival (RFS) analysis revealed that the high expression of CHAF1B was associated with poor RFS, poor one-year, three-year and five-year RFS (HR: 1.92, P = 8.6E-07; HR: 3.07, P = 2.9E-07; HR: 2.14, P = 1.5E-05; HR: 1.94, p = 7e-05).

Moreover, the relationship of the expression of CHAF1B and the HCC patient clinico-pathological variables were also analyzed. The results revealed that the high CHAF1B group had a higher ratio of patients in the advanced American Joint Committee on Cancer Tumor Stage Code (AJCC) stage (33.6 *vs.* 21.4%, p = 0.02) and the advanced neoplasm histologic grade (54.1 *vs.* 27.6%, p < 0.001) when compared with the low CHAF1B group, and more patients in the former group died (43.4 *vs* 31.3%, p = 0.022) (**Table 3**).

**Table 3:** The association between CHAF1B and clinico-pathological variable in TCGA.

Variables	CHAF1B mRNA expression			
		High (n = 122)	Low (n = 243)	p-value
Gender	Male Female	73(59.8%) 49(40.2%)	172(70.2%) 71(29.2%)	0.036
Tumor status	With tumor Tumor free NA	44(36.1%) 70(57.4%) 8(6.6%)	65((26.7%) 160(65.8%) 18(7.4%)	0.189
AJCC stage	T1/T2 T3/T4/T5 NA	81(66.4%) 41(33.6%) 0	190(78.2%) 52(21.4%) 1	0.02
Neoplasm histologic grade	G1/G2 G3/G4 NA	55(45.1%) 66(54.1%) 1	172(70.8%) 67(27.6%) 4	<0.001
Living status	Alive Dead	69(56.6%) 53((43.4%)	167(68.7%) 76(31.3%)	0.022

A univariate Cox analysis revealed that the influence of the advanced AJCC stage (HR: 2.483, p < 0.001) and the high level of CHAF1B expression (HR: 1.737, p = 0.002) on the OS were unfavorable. After adjusting for other factors, a multivariate analysis showed that the advanced AJCC stage and high CHAF1B expression were independent risk factors for HCC patients, with HR: 2.329, p < 0.001 and HR: 1.534, p = 0.019, respectively (**Table 4**).

**Table 4:** Univariate and multivariate Cox regression analysis of variables associated with the OS of HCC patients in TCGA.

Variables OS	Univariate analysis			Multivariate analysis		
	HR	95%CI	p	HR	95%CI	p
Male *vs* female	0.805	0.565–1.148	0.232			
AJCC stage T3/T4/T5 *vs* T1/T2	2.483	1.746–3.531	<0.001	2.329	1.629–3.328	<0.001
Neoplasm histologic grade G3/G4 *vs* G1/G2	1.114	0.776–1.599	0.559			
CHAF1B expression High *vs* low	1.737	1.221–2.473	0.002	1.534	1.073–2.191	0.019

### PPI Network and Enrichment Analysis of CHAF1B

**Figure 5:**
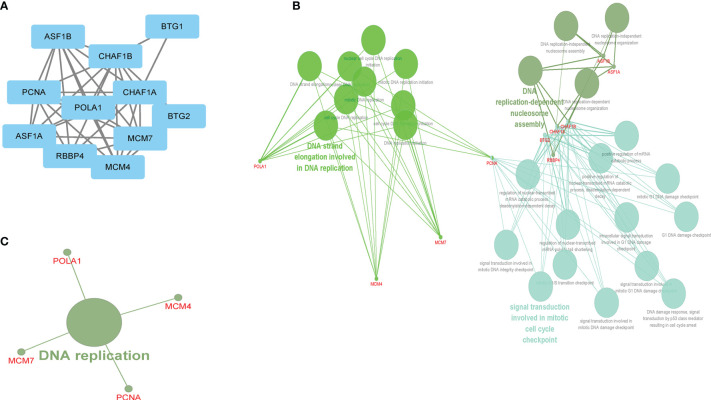
The CHAF1B interacted genes and GO-BP and KEGG enrichment analysis. PPI **(A)**, GO-BP **(B)**, KEGG **(C)**.

MCM7, MCM4, POLA1, CHAF1A, PCNA, ASF1B, RBBP4, ASF1A, BTG2, and BTG1 were significantly interacted with CHAF1B based on PPI network findings (**Figure 5A**). According to the ClueGO plug-in, the GO-BP and KEGG pathways revealed that these genes mainly functioned in DNA strand elongation (which is involved in DNA replication), DNA replication-dependent nucleosome assembly, and signal transduction involved in mitotic cell cycle checkpoints and were significantly enriched in the DNA replication pathway (**Figures 5B, C**).

## Discussion

Liver cancer ranks second in cancer-related mortality and sixth in cancer morbidity globally. China now reports more than half the world’s newly diagnosed cases and deaths (19). Until now, there have been no reliable and effective screening methods for HCC patients who are in the early stage (7). Therefore, it will be helpful if useful biomarkers could be isolated from a body fluid of those at high risk or even in current HCC patients.

Autophagy can be active in the stress response that interferes with the balance of the intracellular environment due to factors such as endoplasmic reticulum stress, hypoxic conditions, pathogen entry anticancer drugs, nutrient and growth factor deficiencies, and low adenosine triphosphate levels (20). Autophagy maintains cellular homeostasis in eukaryotes by recycling and degrading proteins and organelles, which can help in the survival and proliferation of a variety of tumors. Although previous researches have explored the correlation between individual ARGs and HCC, we were the first to analyze all ARGs in-depth. Moreover, the potential molecular mechanisms of CHAF1B in HCC patients has not been investigated.

In this study, we evaluated the expression of 531 ARGs in HCC patients. Based on multi-step selection and validation, CHAF1B not only has a high diagnostic value in differentiating HCC patients from healthy controls but may also be a newly autophagy-related biomarker in HCC, which was significantly associated with the survival of HCC patients. CHAF1B is the p60 subunit of CAF-1 and the central factor of chromatin assembly after DNA repair and synthesis, which is located in Chromosome 21 (21). Consistent with our findings, previous studies have shown that an elevated CHAF1B level was closely associated with a poor prognosis of melanoma (22), prostate cancer (23), salivary gland tumors (24), nasopharyngeal carcinoma (25), breast cancer, and high-grade glioma (26, 27). Our GO-BP and KEGG analysis revealed that CHAF1B was significantly enrich in the mitotic cell cycle checkpoints. Moreover, recently researchers have reported that CHAF1B was associated with cell proliferation, cell apoptosis, and cell cycle arrest (28). However, the exact mechanisms of CHAF1B in HCC still remain unclear, and we indicated that CHAF1B may encourage the development of HCC by affecting the cell cycle progression and autophagy.

Moreover, this study revealed that CHAF1B may play an important role in DNA strand elongation (which is involved in DNA replication), DNA replication-dependent nucleosome assembly, and the DNA replication pathway. In eukaryotic cells, chromosomes encode epigenetic information and regulate the genomic stability. The key process that affects epigenetics is the assembly of nucleosomes, which is the basic structural and functional units of chromosomes. During the cell cycle S phase, DNA uses histones to assemble nucleosomes, a process known as DNA replication-dependent nucleosome assembly (29, 30). Additionally, through the nucleotide excision repair system, CHAF1B can repair DNA damaged by ultraviolet radiation (31). Cancer cells are characterized by active DNA replication and malignant proliferation (32). Therefore, we implied that CHAF1B may be important in the proliferation of HCC patients.

This research has some limitations. Firstly, the clinical verification cannot be conducted. Secondly, because of the limited research conditions, the experiments could not be performed presently. However, we found that the autophagy-related gene CHAF1B is a relevant prognostic and diagnostic biomarker in hepatocellular carcinoma.

## Conclusion

This study supported the inference that the RNA and protein expression levels of autophagy related gene CHAF1B are highly elevated in HCC patients and are also related with poor survival and a more advanced tumor stage in HCC patients. Moreover, CHAF1B is an independent risk factor for HCC. Additionally, CHAF1B plays an important role in DNA replication. Hence, we assumed that CHAF1B may not only play an important role in autophagy, could be a potential novel prognostic and diagnostic biomarker of HCC, but also is vital for the proliferation of HCC cells. Therefore, the molecular mechanisms of CHAF1B in tumorigenesis and development may provide new insights for HCC prevention and prognosis.

## Data Availability Statement

The original contributions presented in the study are included in the article/supplementary material. Further inquiries can be directed to the corresponding author.

## Author Contributions

ZZ and YZ designed the study, designed the figures and tables. WM contributed to the statistical analysis, wrote and corrected the article. All authors contributed to the article and approved the submitted version.

## Conflict of Interest

The authors declare that the research was conducted in the absence of any commercial or financial relationships that could be construed as a potential conflict of interest.

## Abbreviations

HCC, hepatocellular carcinoma; ARGs, autophagy-related genes; AUC, the area under the curve; DEARGs, differently expressed autophagy-related genes; CHAF1B, chromatin assembly factor 1, subunit B; ROC, receiver operating characteristic; OS, overall survival.
